# Cloth face mask fit and function for children part one: design exploration

**DOI:** 10.1186/s40691-022-00287-8

**Published:** 2022-05-15

**Authors:** Jenny Leigh Du Puis, Lauren Forstenhausler, Katarina Goodge, Mona Maher, Margaret Frey, Fatma Baytar, Huiju Park

**Affiliations:** 1 Cornell University, T-41 Human Ecology Building, 37 Forest Home Dr, Ithaca, NY 14853 USA; 2 Cornell University, 255 Human Ecology Building, 37 Forest Home Dr, Ithaca, NY 14853 USA; 3 Cornell University, 150 Human Ecology Building, 37 Forest Home Dr, Ithaca, NY 14853 USA; 4 Cornell University, 112 Human Ecology Building, 37 Forest Home Dr., Ithaca, NY 14853 USA; 5 Cornell University, 235 Human Ecology Building, 37 Forest Home Dr, Ithaca, NY 14853 USA; 6 Cornell University, 133 Human Ecology Building, 37 Forest Home Dr., Ithaca, NY 14853 USA; 7 Cornell University, 131 Human Ecology Building, 37 Forest Home Dr, Ithaca, NY 14853 USA

**Keywords:** Children’s face masks, COVID-19, Mask design

## Abstract

Commercially available children’s cloth masks range widely in material type and fabric structures, methods of construction, layering, and shape, and there is a lack of sizing systems, anthropometric data or guidelines specifically targeting the fit assessment and design of cloth face masks for children 4-6 years old. To better identify and understand the cloth face mask fit and functional needs of children ages 4-6 years old, the researchers embarked on interdisciplinary in-depth study to investigate commercial market offerings of children’s face masks, identify consumer perspectives, and explore mask design improvements through design research. By triangulating results from survey feedback, commercial market content analysis, and wear trial observations, the researchers were able to identify important design criteria that can be used in the improvement of children’s cloth face mask design: size, comfort, dexterity, movement, and thermal comfort. These criteria were used to iteratively develop new mask prototypes involving a 3D printed head form, traditional sewing and hand patternmaking skills, and the creation of multiple mask versions to explore the design criteria listed above. The designs were interpreted through Bye’s (2010) Problem-Based Design Research (PBDR) framework, which identifies common design research practices in the field on a spectrum and situates PBDR as a process centered on a problem as impetus for design through which artifacts are developed.

## Introduction

As of late October 2021, COVID-19 rates in the US for children under 12 years of age have risen to over 2.85 million cases, with 409 deaths (Centers for Disease Control and Prevention, n.d.-b), and though vaccines are readily available for those aged 12 years old and up, they are not yet approved for use in the younger populations. Further, the Delta variant of the COVID-19 pandemic is more transmissible and affects children and others who are unvaccinated in higher numbers than did the original virus strain (Unicef, 2021), and the number of children affected by a condition associated with COVID-19 called *multisystem inflammatory syndrome in children*, or MIS-C, has increased to over 5.2 thousand, with 46 deaths (Centers for Disease Control and Prevention, n.d.-a). Outside of vaccinations, methods to help prevent the transmission of COVID-19 include social distancing, hand washing, and the wearing of face masks. Because medical face masks such as N95 respirators are needed for health professionals and fluctuations in the supply chain impact the availability of disposable surgical masks (Gereffi, 2020), it is incumbent upon the general population to rely on cloth face masks as an alternate option for covering the nose and mouth. Cloth face masks are a convenient, cost-effective option that can be washed and re-worn, and made at home or purchased commercially (De Silva et al., 2020).

The Centers for Disease Control and Prevention (CDC) advise the wearing of cloth face mask use in individuals over the age of 2, and for those who the wearing of masks would not impede medical issues. With the current vaccination age set at 12 years old in the US, children ages 2–11 must use masks as a deterrent to transmission, and particularly while indoors during school (Centers for Disease Control and Prevention, n.d.-c). Limitations surrounding children’s face mask use includes their developing dexterity and subsequent need for adult assistance with donning or doffing, discomfort due to extended wear, and difficulty in breathing while wearing the face masks (Seo et al., 2017). Children ages 4–6 years old have limited ability to carefully handle social distancing, and difficulty in dexterity for the donning/doffing of face masks which can lead to self-contamination and spread of infection in the presence of COVID-19. Compared to older children, young children have increased vulnerability to respiratory epidemics due to the relative weakness of their immune systems and significantly higher respiration rates per minute than adults, that can cause airborne viruses to linger (Kim et al., 2010) and are vulnerable to possible infection while spending time in daycare, schools, and during activities with physical and verbal interactions with other children. Unlike adults, children in this age range cannot adjust the fit of face masks independently or easily, and this population needs frequent adult support for donning, doffing and adjusting of face masks multiple times a day which can increase the possibility of self-contamination and transmission.

In addition, there is no anthropometric data available to be used for the design and sizing of face masks for children in this age range. According to literature review, the fit of a facemask is as important as effectiveness of its filtering materials to protection, and that children ages 4 to 6 years old were identified as particularly in need of assistance with face masks due to a general dearth of information surrounding their specific anthropometries and functional requirements. While adults can easily adjust mask fit using the metal nose bridge or ear elastics, it is extremely difficult to adjust the fit of children’s masks due to their smaller features, which can result in the inhalation of contaminated air such as through the open space around their nose or gaps around the sides of the face or under the chin. Throughout the COVID-19 pandemic there has been a surge in home or community production with those possessing the ability to sew taking on labor required to produce cloth face masks (Snyder et al., 2020). While those with sewing abilities may be able to create or alter cloth face masks for children, many others rely on commercially available options. Vague sizing across commercially available masks for children can create difficulty in sourcing well-fitting options, stemming from a lack of standardization in fit, sizing, materials, and consideration for functional needs of the young wearer.

### Study design

Commercially available children’s cloth masks range widely in material type and fabric structures, methods of construction, layering, and shape, and there is a lack of sizing systems, anthropometric data or guidelines specifically targeting the fit assessment and design of cloth face masks for children 4–6 years old. A review of the literature identified a paucity of practical and translative research that can provide the public with comprehensive and clear guidance for the selection of children’s face masks communicating the impact of material types, layering, design, sizing, and fit on the effectiveness of protection and comfort. It was therefore important to explore these areas targeting children ages 4 to 6 years old, as this is an age demographic as-yet excluded from general study and analysis.

To better identify and understand the cloth face mask fit and functional needs of children ages 4–6 years old, the researchers embarked on interdisciplinary in-depth study with a three-pronged approach: (1) investigating commercial market offerings of children’s face masks, identifying consumer perspectives, and exploring mask design improvements through design research; (2) identifying and testing materials and new mask designs for filtration efficiency and air permeability; (3) evaluating anthropometric sizing and fit in the target age range of children ages 4–6 years old. The study is divided into three manuscripts, and this present text represents the perspectives of commercial market offerings, consumer perspectives, and design research outcomes.

The two-part research problem was identified as follows: (1) the lack of clear knowledge, guidance, and standardization about the fit and functional needs of cloth face masks in children ages 4–6 years old, and (2) the lack of well-fitting commercially available cloth face masks for children ages 4–6 years old. The research questions (RQs) guiding the present study are as follows:

RQ1: How do commercially available cloth face masks fit and incorporate functional considerations for children ages 4 to 6 years old?

RQ2: How can cloth face mask design be improved for children ages 4 to 6 years old? The purposes of this multi-method study were (1) to explore the phenomenon to better understand the fit and function of commercial cloth face masks for children and (2) to use qualitative and design research skills to develop improved options for children’s cloth face masks targeted to children ages 4–6 years old. The study addresses the research questions through the following objectives: (1) explore commercially available children’s face masks’ attributes; (2) identify consumer needs for children’s cloth face masks; (3) develop alternative cloth face mask designs for children ages 4–6 years old. Interpreted through Bye’s (2010) Problem-Based Design Research (PBDR) model, the researchers conducted a content analysis of children’s face masks through market research, interpreted survey responses regarding children’s face masks from adults who are either parents/guardians of or work with children ages 4–6 years old, and conducted iterative design research developing mask prototypes to explore alternative and improved designs for children’s face masks.

### Literature review

#### Face mask use

In addition to social distancing, wearing face masks is the predominant guideline for reducing the airborne transmission of the virus that causes COVID-19, SARS-CoV-2. Infected individuals can expel respiratory droplets in a wide range of sizes, but the smaller particles (< 5 μm) are of major importance as they are likely to contain infectious particles (SARS-CoV-2 and other pathogens) and remain suspended in the air for longer residence times (Fennelly, 2020). The protection given by face masks comes from filtration provided through the types and layers of materials used and the way the masks fit to the face over the top of the nose, sides of the face, and at the chin, which help to eliminate air gaps between the wearer and the environment (De Silva et al., 2020). When worn correctly, these masks can help to prevent the spread of the virus. Other commercially available devices worn on the face to help in protecting the wearer include neck gaiters and face shields, however, unclear messaging and conflicting study results have caused confusion surrounding the use of these items. Face shields provide a clear barrier for the wearer, but their design allows for droplets and aerosols to pass around their edges through large air gaps and should be used in conjunction with other measures such as face masks (Lindsley et al., 2021).

### Children’s cloth face masks

Eberhart et al. (2021) explored children’s face masks through a review of the literature and in doing so discovered that there was such little information available on the topic that a second round of literature review was necessary, calling for further studies targeting different age groups. Mickells et al. (2021) explored adherence to face mask use in elementary schools and found it to decrease later in the day or at the end of the week and was more challenging for the students after periods of independent work or time such as lunch and recess. They further learned more specifics related to children’s mask wear discomfort, including difficulty breathing, students feeling hot, and “baseline poor fit of the masks as well as masks becoming wet and stretched which led to worsening fit over time” (Mickells et al., 2021, p. 559). These findings reinforce the need for (1) study in this area and (2) importance of design considerations related to comfort and fit.

### Children’s face mask design

In February 2021 the CDC issued a report on ways to enhance the fit of face masks for better performance, including double-masking (1 surgical mask next to the face with 1 cloth mask on top) and modifying surgical masks to better fit the face to reduce air gaps including through knotting the ear elastics and folding excess gapping mask edges inward (Brooks et al., 2021). A similar process was undertaken by a team of doctors in Italy with researchers adjusting surgical face masks to fit children’s faces (Lubrano, 2021). While these two studies specifically utilize surgical masks, a common thread between the two is the necessary adjustment of a rectangular pleated mask to better fit the contours of the human face and reduce air gaps or adjust to a more appropriate size, as with children. This adjustment exemplifies the importance of fit to the face with masks overall, and specifically the popular pleated style.

### Problem-based design research

**Fig. 1:**
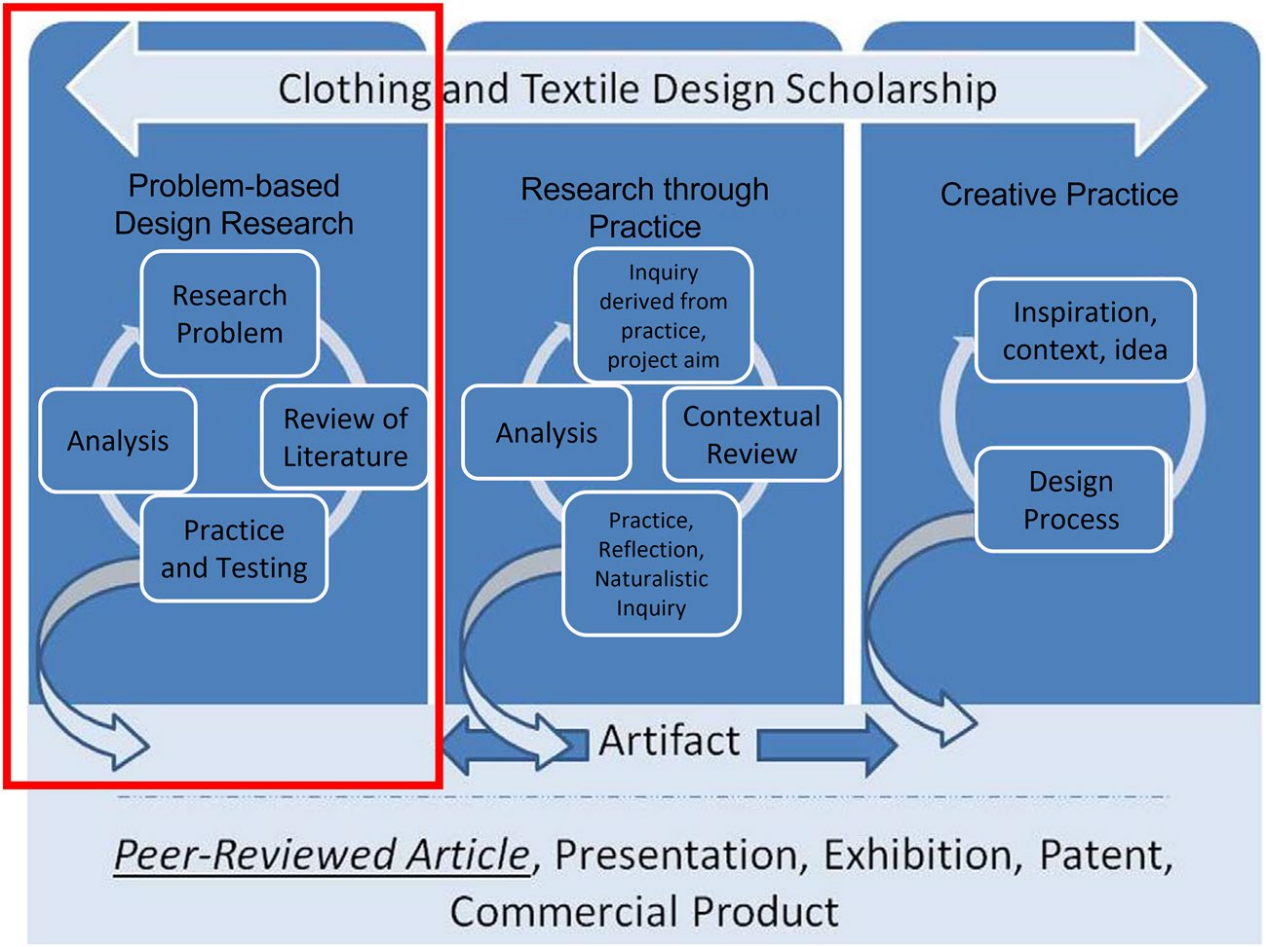
Framework for Clothing and Textile Design Scholarship by Bye, (2010). Note. The red box indicates the PBDR model used in the present study

In Bye’s (2010) Problem-Based Design Research (PBDR) framework (see Fig. 1), the identification of a problem serves as the root of the design process for the exploration and development of artifacts. This is accompanied by a review of literature and the development of prototypes, which are analyzed in relation to the original problem. Through this process, multiple artifacts are created. In this present study the artifacts were the masks themselves along with their patterns, notes, and photographs. Data gathered from these items were compared with results from the market research content analysis and the survey responses to create a circular process for the development of cloth face mask improvements and modifications for children ages 4–6 years old.

## Methods

The present research study was comprised of multiple parts: identifying consumer perspectives related to face masks in children, investigating commercial market offerings of children’s face masks, and exploring design improvements. Methods undertaken include survey research with subjective and objective responses, a market research content analysis, wear observations, and design research through iterative prototyping. Results of the survey research, content analysis, and wear observations were triangulated with subsequent conclusions used as design criteria for mask modifications and improvements in new designs. Data acquired through the study were analyzed through a variety of techniques including content analysis and visual observations, identification of emergent themes and descriptive statistics of quantitative survey responses, pilot wear test observations of commercial masks, and review of artifacts developed through PBDR (Bye, 2010) including mask patterns, prototypes, photographs, and notes.

### Online survey

After IRB approval was gained from the researchers’ university, a Qualtrics survey was distributed online by advertising the study on social media. The survey included multiple choice and open-ended questions. Not all participants answered every question as respondents were able to self-select into sub-categories, which helped to customize the survey to the participants and showed only questions that best aligned with their experiences. Participants were asked to select any modifying factors that would affect the children’s mask wearing (e.g., glasses, disabilities, or special needs). Additional questions included experience with different types of masks and mask fastenings, and subsequent ratings of preference, comfort, donning/doffing, and fit, as well as issues they may have faced with their mask of choice. Mask composition-related questions included preference of materials, number of layers, inclusion of filter pockets, bendable metal nose bridges, reversibility, and visual surface design. Descriptive statistical analyses were conducted in Excel.

### Market review and selection of test masks

A content analysis of commercially available children's face masks was conducted for market research. Google searches of "children's face masks" and "kid face masks" yielded curated lists from *Vogue* (Schama, 2020), *People* (Warner, 2020), *Today* (Boan & Ortiz, 2020), *Good Housekeeping* (Sachs, 2020), and the *She Knows* blog (Weiss, 2020) for a total of 113 unique face masks, of which 30 were recommended by two or more sites. Screenshots were captured of each mask listing and PDF reference sheets created for each of the five curated lists, including images of the masks and listing text content. A Microsoft Excel comparison chart categorized each mask's attributes including style, fastening type, number of fabric layers, material properties, and type of surface design. These attributes were compared within their categories to find the overall most common children’s commercial mask attributes as found on the five original curated lists (see Table 1).

**Table 1:** Market research content analysis results

Style				Fastening			
		%	*n*			%	*n*
	Shaped	57%	64		Stretch Ear Loops	67%	76
	Pleated	26%	29		Adjustable Ear Loops	24%	27
	Stretch	20%	18		Behind Head	9%	10
	Gathered	3%	3		Ties	5%	6
	Gaiter	0.9%	1				

Materials			Surface design			Layers			Composition		
	%	*n*		%	*n*	#	%	*n*		%	*n*
Cotton	78	88	All-over print	57	64	1	4	5	Woven	36	41
Bamboo	0.9	1	Solid Color	38	43	2	56	63	Knit	20	22
Spandex	11	12	Centered Print	13	15	3	16	18	Unknown	16	18
Polyester	21	24				4+	0.9	1			

### Wear trials, user observations and fit assessments

**Fig. 2:**
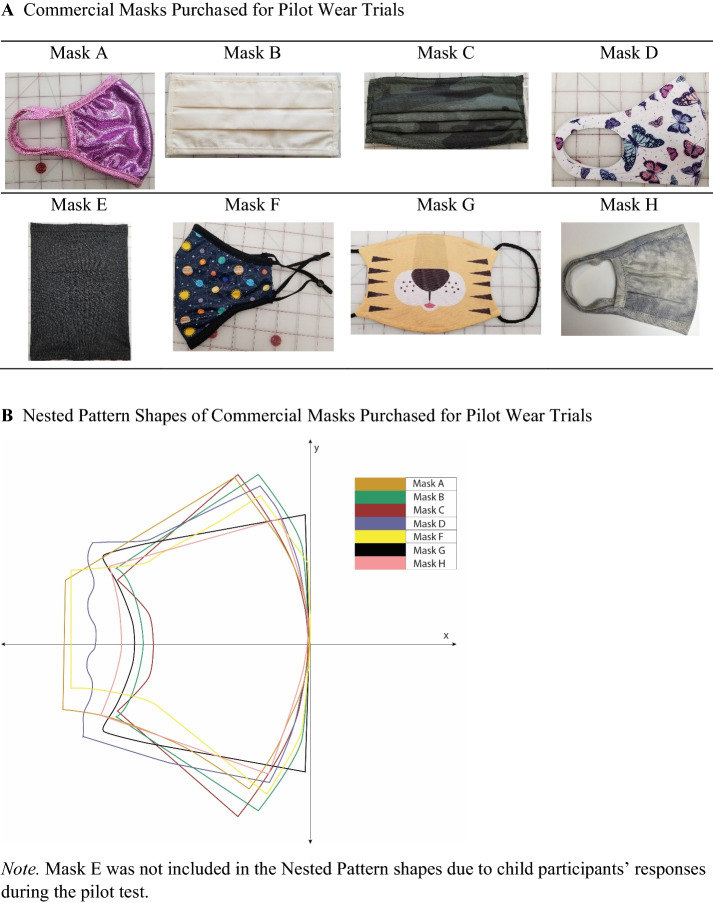
Commercial Masks Purchased for Pilot Wear Trials & Nested Pattern Shapes

After review of these attributes, eight masks (see Fig. 2A) were purchased for observation of users’ wear trials, investigating fit issues and exploring fabrics and overall fit. The selection criteria of these eight masks included fiber/fabric types and number of layers, style, size adjustability, and use of filters, with masks purchased to represent a variety of these attributes. Of these eight test masks, six were recommended by more than one curated list and the remaining two masks were selected to round out the testing across attribute categories. The test masks were further evaluated for design and construction techniques and pattern shapes were traced and digitized to explore shape comparisons (see Fig. 2B), and sizing was measured by hand with a tape measure. Finally, pilot wear tests were conducted with each of the eight test masks.

Due to the difficulty in recruiting children as participants during the ongoing pandemic with adherence to lockdown recommendations, the overarching need for safety and the limited abilities of children to follow a multi-step evaluation process for feedback through numeric ratings, three children ages five (*n* = 2, one boy and one girl) and six (*n* = 1, boy) years old from the researchers' families wore a selection of face masks indoors and outdoors to ascertain real-life responses and observations by members of the research team. Though recognized as a limitation in the research methods, the observations of masks on these three children provided valuable feedback when placed in conversation with survey results. They were interpreted through the lens of the PBDR framework (Bye, 2010), reflecting common issues with the fit and wear of commercially available children’s face masks and providing insights into ways to improve existing mask designs. With IRB exemption approved from Cornell University (IRB No.: 2008009743), observations were recorded using memos and photos, focusing on (1) noticeable/visual fit issues, (2) ease of donning and doffing, and (3) fogging of eyeglasses, which indicates air gaps around the nose.

### Design research

**Fig. 3:**
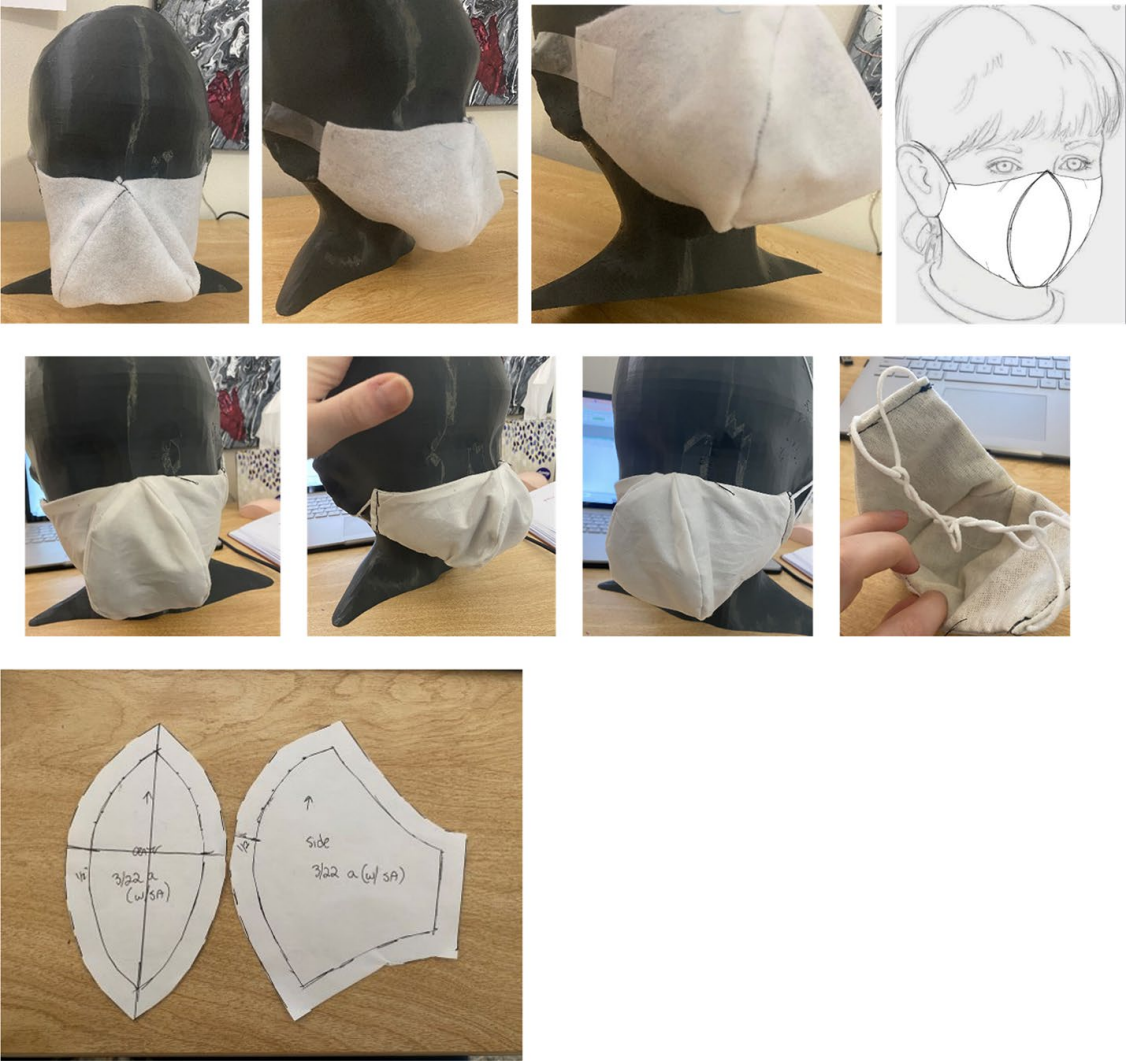
“Orb” Mask Sketch and Prototype Iterations

**Fig. 4:**
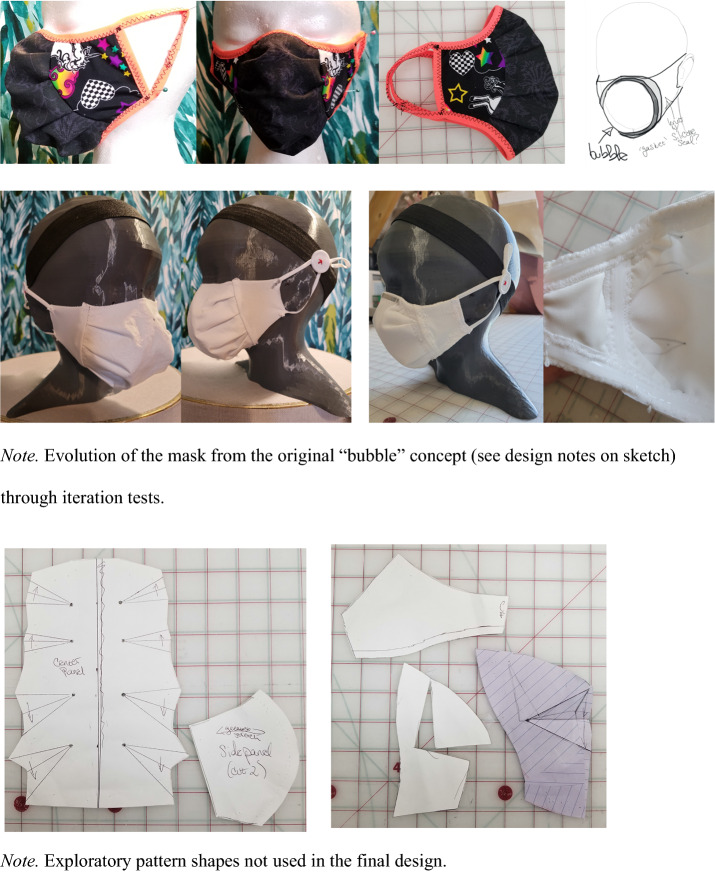
“Lobster” Mask Sketch and Preliminary Iterations. Evolution of the mask from the original “bubble” concept (see design notes on sketch) through iteration tests

Following the PBDR framework (Bye, 2010), design-centered information gained from the triangulated survey findings, market research content analysis, and wear trial observations were used as integral knowledge for the design and refinement of two prototypes to improve children’s face mask designs. These prototypes were developed by the apparel design student researchers and evolved through refinements and discussion with the rest of the research team. The two mask prototypes were named the “Orb” (see Fig. 3), and the “Lobster” (see Fig. 4), based on their shapes’ resemblances to other objects. The “Orb” mask utilized shaping techniques to provide greater space for breathing around the nose and mouth. However, through prototyping it was discovered that the overall silhouette had issues with collapsing at the center, and that the prototype was bulky due to the construction techniques used. The “Lobster” mask included considerations for expansion of the mouth in dynamic movement through the use of pleats and stretch side panels, and did not face the same concerns with bulk or misshaping. As such, it was decided by the research team to move forward with the “Lobster” design for the rest of the research study.

## Results

### Online survey and market research

Of the 93 total responses collected for the online survey, 27 did not answer questions beyond the basic demographics or otherwise failed to finish the survey and thus the remaining 66 responses were deemed usable*.* Respondents were parents/guardians (70%), teachers (19%), coaches/hobby instructors (6%), and healthcare workers (5%) who had experience helping children 4–6 years old with their face masks. Results of the online survey indicate issues with the fit, size, wear, and comfort of masks. Table 2 shows the categories, quantitative responses, and examples of qualitative feedback as answered by the 55 respondents who provided feedback on mask fit.

**Table 2:** Online survey mask feedback, total n = 55

Category	%	*n*	Related qualitative comments
Mask too loose and slips down face	69	38	“If the ear strap is not tight enough, I have to help them twist it to tighten it” “Movement causes adults’ masks to dip below their noses, children are no different” “I am concerned that extended wear will cause discomfort behind the ears” “Most store-bought masks are too big for smaller children and fall off the nose” “Too close to mouth makes it enticing to chew” “…tight fitting masks with multiple layers are hard to breathe through”
Ear straps too loose	64	35	
Mask does not stay in place	45	25	
While talking	33	18	
During everyday physical activity	31	17	
During strenuous physical activity	38	21	
Discomfort on back of ear	44	24	
Mask too large	40	22	
Length too long on face	31	17	
Width too wide on face	25	14	
Mask too close to mouth	22	12	
Too difficult to breathe through	22	12	
Difficulty fastening behind head	16	9	
Length too short on face	11	6	
Mask too small	11	6	
Width too narrow on face	9	5	
Mask too tight on nose	7	4	
Mask fogs up glasses	5	3	

69% of the respondents indicated that commercial masks were too loose and slip down the face, while for the 64% of the respondents’ ears straps were too loose. Other most frequently reported issues were that the mask doesn’t stay in place (45%)—especially during strenuous physical activity (38%) and while talking (33%); discomfort on back of the ear (44%); and mask too large (40%). Further qualitative feedback from the survey provided deeper insights into the difficulties that arise with face mask use in younger children. Some common themes that arose include heat and dampness inside the mask, slippage due to movement or ill fit, the need for alterations or adjustments to improve fit, the need for stowage (such as a neck strap) when temporarily removed, and issues related to the size and sensitivity of children’s ears.

Some direct quotes from the survey feedback include the following. Regarding size and fastenings, “Almost always more difficult if any tying is required. Most store bought masks are too big for smaller children and fall off of the nose.” Pertaining to thermal comfort: “Children get hot under the mask. Masks get damp/wet inside and it makes them less effective and uncomfortable.” And finally, related to size and movement: “Masks that are too loose or tight (so they’re uncomfortable staying on) or not staying on the nose while they talk.” Due to their young age and developing motor skills, children may not have the dexterity required for tying face masks or reaching behind their ears to secure ear loops. There were also several comments that mentioned long hair complicating the donning process and children chewing on the inside of their masks, especially if fitted too close to the mouth.

In the market research content analysis results, comparisons of listings yielded identification of the following physical mask attributes that were considered in the proposed mask designs: style, fastening type, number of layers, materials, fabric structure, and surface design (see Table 1). Add-ons with lower frequencies included the specification of an enclosed metal nose piece (16%, *n* = 19), inclusion of a filter pocket (32%, *n* = 37), and indication that the mask was reversible (7%, *n* = 9). The comparisons among these categories helped to identify the most common configurations from the lists. Collectively, the most common mask would be the shaped style (57%) with stretch ear loops (67%), comprised of 2 layers (56%) of woven (36%) cotton (78%) with an all-over print (57%).

### Observation of wear trials of the commercial face masks

**Fig. 5:**
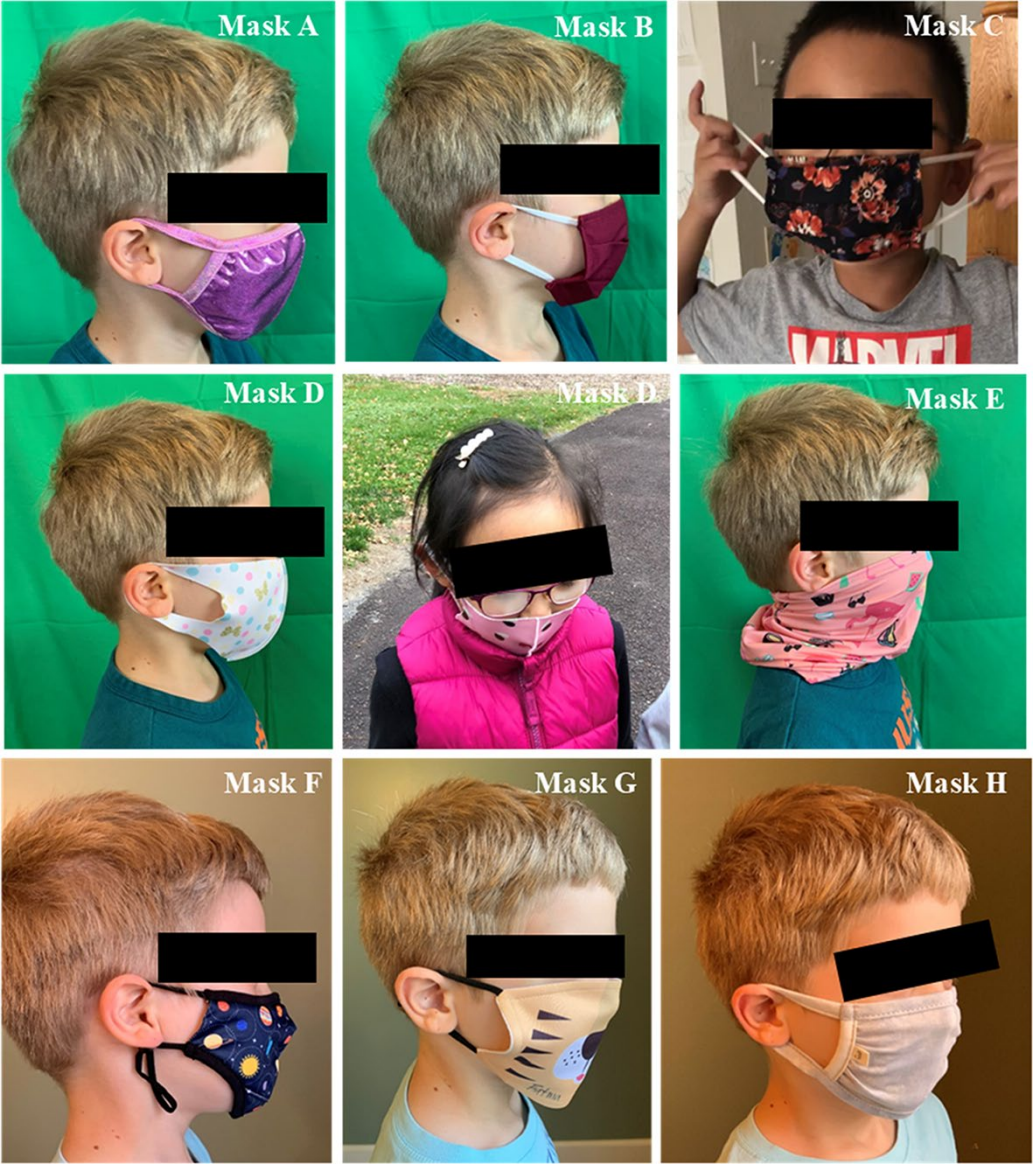
Observed Wear Trials and Fit Issues. Exploratory pattern shapes not used in the final design

The eight selected face masks were examined for design and construction techniques by the research team, and for use in wear trials by the child participants. The researchers’ university Institutional Review Board exempted the protocol for observing the eight purchased masks as worn by child participants, and the following observations of mask-related issues were made by members of the research team (see Fig. 5). Mask A was constructed of stretch fabric with a center front seam and stretch binding stitched around the perimeter of the mask, which creates a gathered shape that cups around the nose and mouth. However, this mask presented issues with the size of the non-adjustable ear loop being too small, which can cause the mask to pull tightly to the face and create tension and uncomfortable strain behind the ear. Masks B and C were non-stretch pleated masks found to be difficult to speak through due to tightness against the face, the label on the mask rubbed against the wearers’ skin, and the ear elastics were too small, causing discomfort. Mask D was a single-layered stretch shaped mask with built-in ear loop cutouts and a center front seam. This mask was reported positively by two of the child participants (age 5) regarding fit and placement, and negatively by the third (age 6), who was found to have issues with mask slippage out of place. Observations by the researchers include gaps around the edges of the mask and fogging of glasses, indicating issues with fit. Mask E, a gaiter style, was identified by all three participants as the most difficult to wear and use, and resulted in extra fabric bunched around the neck, which was an additional discomfort factor. Mask E was made of a tight and long knit fabric tube, which caused difficulty in donning due to the need to firmly pull the mask first down over their heads and then up to cover their small noses and mouths, which was challenging due to the limited dexterity of this age group. Mask F was a shaped style with a dart for the nose and separate piece of fabric for the chin, joined with a seam designed to cup around the face. Ear elastics were adjustable, which permitted for a more custom fit to the wearer. Mask G was designed as a flat, 2-dimensional panel that when worn is wrapped around a 3-dimensional form (the human face) with no shaping involved such as darts or gathers. This technique leaves large gaps at the edges of the mask, allowing for more air to pass around to the nose and mouth, and the elastics create tension on the ears causing discomfort. Mask H was constructed of a single layer of stretch fabric, gathered around the edges with a stretch binding in a technique similar to Mask A. However, due in part to differences in sizing and shaping, Mask H was found to be very tight across the face and pulled on the ears.

#### Wear trial themes

Shaped masks such as D and F fitted closer to the face, reducing air gaps. However, while mask D fitted closer to the face, it and masks E and H were comprised of single layers of fabric, which is one layer fewer than what is recommended by the CDC (Centers for Disease Control and Prevention, n.d.-d). Poor fit was the most dominant issue in six test masks out of eight. As shown in Fig. 4, test masks B and C (pleated), D (shaped), and G (stretch) showed obvious gapping on the side, which can cause the air near the face to flow in and out freely without filtering through the face mask. The test masks B and H were too tight around the nose and mouth which may cause difficulty in breathing and speaking, causing discomfort as reported by all three participating children. Other observation findings showed that elastic ear loops can cause the mask to flip inside out if pulled too hard when adjusting for eating, drinking and resting. Fogging was observed in all test masks in two participating children who wore glasses, leading to poor visibility and anxiety, in wear trials performed outdoors in November 2020 at temperatures colder than normal body temperature (~ 98˚ F) which helped identify basic issues with the commercial mask designs. Mask donning and doffing was also an issue compounded by glasses wearing, as mask fastenings tangled with the arms of the glasses behind the ear. Mask F was most preferred due to its fitted shape around the nose, mouth and under chin coverage, and space-themed surface design.

### Design criteria

Results from the online survey and observations of the commercial mask wear trials consistently indicate that there is a concern of poor fit in children’s cloth face masks, which can cause discomfort or result in poor filtration with gapping and slipping out of the proper position, contributing to a higher possibility of adjustment of the fit, and its resultant cross contamination and ineffective protection. Common themes across children’s face masks were related to issues with size (too small and too large), comfort (tension across the face, pulling behind the ears), dexterity (handling fastenings behind the ear and general donning/doffing), movement (masks slipping out of place when talking or being active), and thermal comfort (heat, dampness). These findings are echoed in Mickells et al. (2021), reinforcing the need for in-depth investigation into improvements for children’s cloth face masks. To address these needs, the above resultant issues from the survey results and wear observations were distilled into design criteria themes that were compared to results from the market research content analysis, and translated through experiential knowledge by the apparel design members of the research team. Following Bye’s (2010) PBDR framework, iterative prototyping was conducted to develop improved cloth face masks for children in the 4–6 year old age range.

#### “Lobster” mask design

**Fig. 6:**
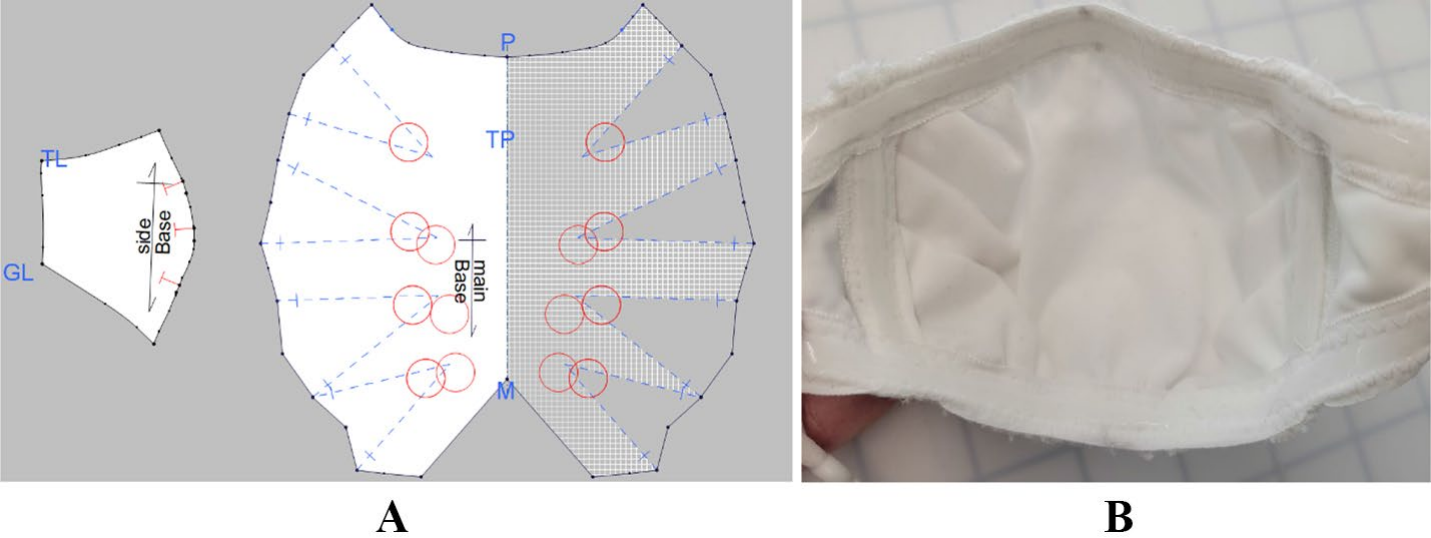
“Lobster” Mask Design **A** Finalized Patterns and **B** Inner Details

Incorporating the design criteria of size, overall comfort, dexterity, movement, and thermal comfort, the “Lobster” mask was used as a sample. By following problem-based, iterative design methods, the “Lobster” mask was comprised of three contoured pattern pieces, featuring a layered center front panel with pleats that was designed to expand for comfort while increasing filtration surface area (Salter, 2021) and two side panels made of nylon tricot that allow for movement of the jaw. Similar to the concept behind a “mask fitter”, which is a device that sits on top of existing masks and acts as a frame to help seal the mask’s edges (Meiller, 2021), the “Lobster” mask was developed with silicone elastic around the perimeter of the edges and in between the stretch panels on the sides of the face and the central pleated filtration panel. The silicone elastic provides a seal around the mask that also enables movement and comfort through stretch (see Fig. 6) and was included due to experiential knowledge in functional apparel design. The use of silicone in mask design was later also found to be echoed in Salter (2021). In the “Lobster” design, these strips line the edges of the mask, including the top, bottom, and the side front seams connecting the stretch side panels to the center front pleated panel, which works to keep the mask in position on the face and also creating a barrier between mask fabric resting against the skin and not actively working as a filter (Salter, 2021) and the center front pleated filtration panel. A bendable metal nose piece was affixed to the top of the mask for customization of fit over the bridge of the nose.

#### “Lobster” mask sizing and patterning

Sizing and patterns for the “Lobster” mask were developed through both knowledge of measurements of masks from the commercial market research portion of the study and by draping basic mask pattern shapes on a 3D printed head form, which is a common technique used in apparel design with fabric draped around a form to create basic garment foundation shapes from which more elaborate patterns may be developed. To prepare the head form, a body scan was randomly selected from the Size NorthAmerica dataset for a 6-year-old boy in the normal weight range, which yielded a sample head sized 8R with a head girth of 55.8 cm. The resultant draped mask shapes were used as base patterns for the development and refinement of the “Lobster” mask. Iterative prototypes were placed on the head to identify gaps around the edges or ill-fit, which were in turn adjusted in subsequent patterns and prototype iterations (see Fig. 6).

Because of the difficulty in 3D printing a head with protruding ears, it was necessary to create a system where ear elastics on the mask prototypes would be consistent, and thus a headband of wide elastic with silicone strips was sized to the head, and 1″ buttons attached. This mimics the “ear savers” commonly used by mask wearers during the pandemic, where ear elastics are fastened to buttons on a headband or strip of ribbon to mitigate repeated and lengthy wear of masks that would cause pressure and soreness to the skin behind the ear. Pressure and discomfort behind the ear were also found as a result of the commercial market research mask wear trials, and the survey feedback, indicating the need for fastening consideration. Due to the popularity of ear elastics and the need for adjustability, it was decided to integrate soft stretch ear elastics with silicone adjustment sliders into the mask design, with the intent to alleviate pressure behind the ears and accommodate differing head sizes.

#### “Lobster” mask materials

Selection of materials for the “Lobster” mask yielded a mixed composition of each layer’s fiber content and fabric structure. These were chosen to be polyester interlock/polypropylene nonwoven/polyester interlock based on the results from the commercial mask performance testing (further detailed in the forthcoming second manuscript of this larger research study), the American Association of Textile Chemists and Colorists M14 guidance for adult barrier face coverings (2020), and CDC and World Health Organization (WHO) recommendations. Guidance from the WHO (World Health Organization, n.d.) encourages cloth masks to include three layers: a hydrophobic barrier layer, a middle filtration layer of a material such as polypropylene, and a hydrophilic layer closest to the face. The “Lobster” mask follows these guidelines for the outer and middle layers, but diverges for the innermost layer, which was exchanged for a hydrophobic fabric, which does not keep moisture next to the face thereby minimizing dampness and other thermal discomfort.

Because of (1) the survey feedback indicating that children may chew on the inside of their face masks and (2) the need for balance between fabric type and hand in constructing cloth face masks, the polyester interlock inner- and outermost layers were stabilized with a lightweight, stretchable interfacing called Pellon EK130 Easy-Knit™, sourced through a local fabric store. The polypropylene nonwoven layer was cut to the same size as the outer layer to further mitigate any deformation of the fabric structures during wear, i.e., to eliminate stretch and maintain structure around the nose and mouth.

#### “Lobster” mask fit

**Fig. 7:**
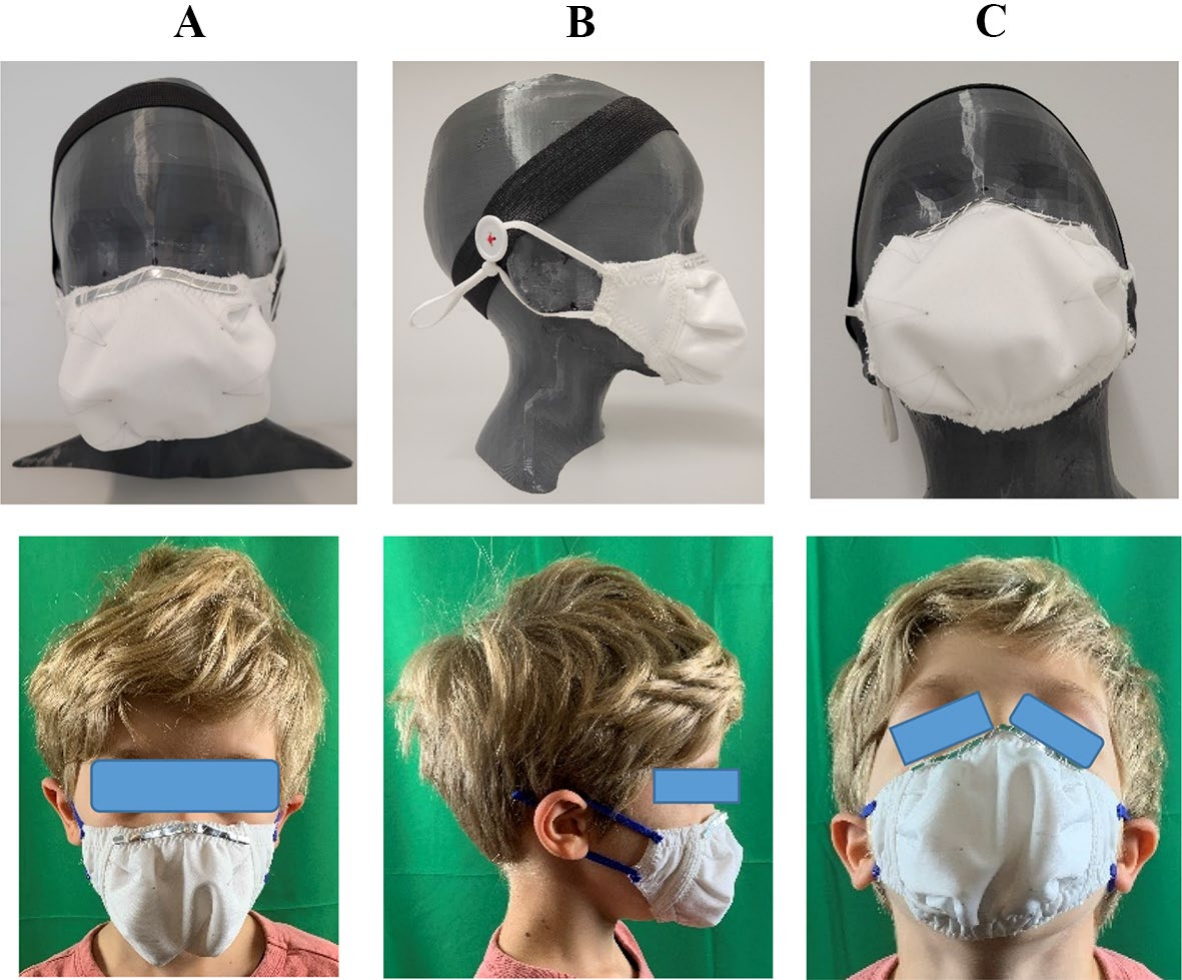
Lobster Mask Fit **A** Front, **B** Side, **C** Detail on 3D Printed Head and 6-Year-Old Child Participant

The fit of the “Lobster” mask was pilot-tested on the 3D printed head form as well as with the 6-year-old participant from the commercial mask wear observations (see Fig. 7). The initial findings revealed that the “Lobster” mask was able to answer some of the needs highlighted in the survey results as well as commercial mask fit observations. Specifically, the mask was more closely sized to the child participant, decreasing issues with the mask being too large or too small; overall comfort was improved through use of the strategic placement of the larger center front panel shaped to create space around the nose and mouth; dexterity through the use of large and soft ear elastics with adjustable sliders for a custom fit; and thermal comfort through layering of hydrophobic and hydrophilic materials intended to keep moisture and dampness away from the face. Movement was accommodated through multiple techniques: strategic placement of stretch panels on the sides of the mask, allowing for opening and closing of the jaw without strain, the inclusion of silicone elastic around the edges to help keep the mask in place by reducing slippage, and the soft stretch ear loops allowing for less pressure behind the ear. Additional findings include improved fit over the bridge of the nose due to the use of a longer bendable metal nose piece, and overall good covering proportionately over the face.

## Discussion

The significance of the research cannot be understated – there are very few studies exploring face masks for children in this age range, and even fewer studies being conducted that investigate cloth face masks from the specific perspectives of design, anthropometry, and fiber science. In developing worn objects for the human body, it is essential to consider factors above and beyond those presented in safety briefings so that the objects (in this case cloth face masks) not only meet basic safety parameters but also consider the human experience of wear, including proper fit, comfort, sizing, and materials. Throughout the inquiry into cloth face masks for children, the research team collaborated across subspecialties, demonstrating the importance of interdisciplinary work of this type. Practical applications for the research outside of the academy include improved understanding for industry of design criteria for developing children’s face masks and a call to action for the refinement of masks for this vulnerable population. Subsequent manuscripts (the forthcoming parts two and three of this manuscript series) will present information related to materials and sizing, with an overall result of expanding the knowledge base in this area not only for academia and industry but the general public as well.

Mask design refinements are important to developing improved fit and comfort, and consumer feedback through survey results coupled with wear trials and market research provides vital information for the mask designer. In the present study, this information was used to iteratively develop cloth face masks for children that incorporated considerations for fit, comfort, sizing, and materials. To address the design criteria identified through the survey responses and wear trial observations, the “Lobster” mask development sought to create a cloth face mask option that would accommodate for size (through use of a representative 3D-printed head sample for base pattern development), overall comfort to reduce tension and pulling (use of pleating to bring the mask away from the nose and mouth creating a pocket), dexterity (use of ear elastics that can be adjusted by an adult for custom fit then easily reused by children), movement (silicone elastic around the edges to reduce slipping and create a seal, as well as more mindful fit to the face), and thermal comfort (integrating a multi-layer lighter weight combination of fabrics).

## Conclusions

Through this study, the authors considered the following research questions:

- RQ1: How do commercially available cloth face masks fit and incorporate functional considerations for children ages 4 to 6 years old?
- RQ2: How can cloth face mask design be improved for children ages 4 to 6 years old?

In response to RQ1, the researchers explored commercial designs available on the market through content analysis, leading to the purchasing of eight masks for evaluation by apparel design members of the research team and wear trial observations by three human subjects ages 5 and 6 years old. Results of these forms of study were triangulated with survey responses to ascertain information related to the fit and functional considerations of commercial mask designs. Conclusions related to RQ1 include that broadly, commercial face masks do not comfortably fit children in the 4–6 year old age range and that improvements are necessary. Further, functional considerations of commercial face masks were distilled through the triangulation of data from the content analysis, survey responses, and wear trial observations to develop essential design criteria for children’s cloth face masks in this age range. These design criteria are size, comfort, dexterity, movement, and thermal comfort, and it is clear through the study results that there exist many issues across commercial masks related to functional considerations for cloth face masks in this age range. To better address these issues, mask designers and manufacturers should more closely consider size, comfort, dexterity, movement, and thermal comfort needs in young children. To address RQ2, the design criteria were used to iteratively develop many design prototypes for children’s cloth face masks. In doing so, the researchers used expertise in apparel design to include such concepts as (1) more appropriate sizing through draping base patterns on a representative 3D printed head form sized using a six year old’s measurements, (2) shaping the center front of the mask to provide more air space around the nose and mouth, (3) using adjustable soft ear elastics for fastenings that can be adjusted by an adult then reused by the child wearer, (4) silicone elastic around the edges and across the side front seams of the mask to reduce slippage while stretch side panels allow for movement, and (5) layering of fabrics that help to improve thermal comfort for the wearer.

An interesting finding over the course of the research project was the evolution of commercial cloth face mask offerings for children. While at the beginning of the COVID-19 pandemic in the US commercial manufacturers offered face masks developed with broad but rather vague guidance, just as knowledge about face masks changed so too did cloth mask designs overall. Given the evolving nature of mask use, availability, and guidelines for wear, since the research discussed in the present text was completed some additional advances have been made, such as different styles of cloth face masks becoming more popular and widespread vaccination of the public. Though children under 12 years of age are still unable to receive the vaccine, it is only a matter of time before approval (Rabin, 2021). For the wear trial observations of commercial masks on children’s faces, a limited number of masks were purchased to represent a range of mask design attributes and were worn by three children for short periods of time. It was also challenging to recruit more children at the time of data collection when the vaccines were not yet available, and there was a wide spread of fear of infection. At the time, there was a restriction on human interactions unless the research was classified as an urgent health care study. Future research should explore the current state of commercially available cloth masks and utilize more masks with a larger sample of human subjects. Although the present research is aimed at ages 4–6 years old, no children aged 4 years old were recruited for the study due to the need to adhere to safety and lockdown protocols, and thus only children ages 5 and 6 years old wore the masks.

The “Lobster” face mask has yet to be evaluated through extensive wear tests. As it is not an item intended for mass marketing purposes, the focus was on identifying ways to improve different aspects of face mask design for young children. After developing a number of prototypes, certain elements of the design have been identified as needing further refinement such as in reducing bulk at the seams between side panels and center front panel, exploring alternatives for silicone application, and expanding the space inside the center front of the mask. Additionally, little focus was placed on the aesthetic of the design, and surface design was indicated in both the survey results and the wear trial observations as a key way to increase the children’s desire to wear the face masks.

## Data Availability

All relevant data and materials are available upon request.

## Abbreviations

CDC: The Centers for Disease Control and Prevention
PBDR: Problem-Based Design Research
